# Chlamydia Detection during the Menstrual Cycle: A Cross-Sectional Study of Women Attending a Sexual Health Service

**DOI:** 10.1371/journal.pone.0085263

**Published:** 2014-01-27

**Authors:** Dana S. Forcey, Jane S. Hocking, Sepehr N. Tabrizi, Catriona S. Bradshaw, Marcus Y. Chen, Glenda Fehler, Jessica L. Nash, Christopher K. Fairley

**Affiliations:** 1 Melbourne Sexual Health Centre, Melbourne, Victoria, Australia; 2 Melbourne School of Population and Global Health, University of Melbourne, Melbourne, Victoria, Australia; 3 Department of Clinical Microbiology and Infectious Diseases, Royal Women's Hospital, Melbourne, Victoria, Australia; 4 Department of Obstetrics and Gynaecology, The University of Melbourne, Melbourne, Victoria, Australia; 5 Central Clinical School, Monash University, Melbourne, Victoria, Australia; Midwestern University, United States of America

## Abstract

**Background:**

We investigated the detection of chlamydia at different stages of the menstrual cycle.

**Methods:**

Electronic medical records for women attending Melbourne Sexual Health Centre between March 2011 and 31^st^ December 2012, who were tested for chlamydia by nucleic acid amplification of high vaginal, cervical, or urinary samples, and who recorded a date of last normal menstrual period (LNMP) between 0–28 days were included in the analysis. Logistic regression was used to calculate adjusted odds ratio (aOR) and 95% confidence intervals (CI) for the association of chlamydia with menstrual cycle adjusted by demographics and behavioural variables. Chlamydia and beta globin load were determined on those with stored samples.

**Results:**

Of the 10,017 consultations that included a test for chlamydia and a valid LNMP, there were 417 in which chlamydia was detected. The proportion of samples with chlamydia was greater in the luteal phase (4.8%, 184/3831) than in the follicular phase (3.4%, 233/6816) both in the crude (OR 1.29 95%CI 1.1–1.6, p = 0.01) and adjusted odds ratio (aOR) 1.4 (95%CI 1.1–1.8, p = 0.004). Among women using hormonal contraception, there was no significant association with the luteal phase of the menstrual cycle (aOR 1.3, 95%CI 0.9, 1.8, p = 0.18). Among women not using hormonal contraception, there was a significant association with the luteal phase (aOR 1.6, (95% CI 1.1–2.3, p = 0.007). The chlamydia load was not significantly different in the 329 positive stored samples in weeks 3 and 4 vs weeks 1 and 2 for any site (P>0.12).

**Conclusions:**

The higher detection of chlamydia detection in the luteal phase of the menstrual cycle in only those not taking hormonal contraception suggest that hormonal factors influence chlamydia detection. The absence of a significantly highly chlamydia load in women during the luteal phase raises questions about the mechanism.

## Introduction

Chlamydia is the most commonly diagnosed bacterial sexually transmitted infection (STI) in Australia [1]. The notification rate of chlamydia continues to rise, and studies over the last decade have also shown that the underlying prevalence of infection is also rising [2]. Measures to control the transmission of chlamydia have largely focused on screening programs that recommend annual testing in individuals less than 25 years of age [3]. Despite implementation of such programs however, a high level of endemic infection persists in many communities. The success of screening programs will depend in part on the sensitivity of the assay that is used to detect chlamydia. One factor that has not been widely explored is the impact of the menstrual cycle on the sensitivity of chlamydia detection assays.

Susceptibility of the endocervix to infection may be temporally related to the stage of the menstrual cycle [4]. Studies have demonstrated that acute salpingitis caused by chlamydia is significantly more common in the earlier stages of the menstrual cycle, particularly within 7 days of the last normal menstrual period (LNMP) [5]. Three subsequent studies conducted in the 1990's using an amplified enzyme immunoassay and/or direct immunofluorescence demonstrated increased chlamydia detection in the latter half of the menstrual cycle [6]–[8]. However, these studies were relatively small and had limited power to adjust for potential confounding factors and not all used the more sensitive amplification assay to detect for chlamydia.

Our study aimed to investigate associations between the detection of chlamydia infection in women and the stage of the menstrual cycle by analysing the electronic patient records from a large sexual health clinic in Australia.

## Methods

### Ethics Statement

The information used and specimens collected in this study were part of routine clinical practice. The IRB gave consent for this information to be used and the specimens to be analysed in this study without the written consent of each patient.

Ethical approval was obtained from the Alfred Human Research Ethics Committee (560/12).

### Study Population & Data Collection

This was a retrospective analysis of computerised records of women who visited Melbourne Sexual Health Centre (MSHC) between March 2011 and 31^st^ December 2012. The MHSC is a large, public STI clinic in Melbourne, Victoria, Australia. The clinic conducts about 35,000 consultations annually and provides free STI testing and treatment.

Upon presentation to the MSHC, all clients are required to fill in a self-administered computer-based questionnaire, which collects information regarding client demographics, the reason for presentation, sexual practices, use of contraception, prior history of STIs, Pap smear and pregnancy history and history of recreational drug use and sex work. The triage nurse records the presence or absence of genital symptoms when the client first arrives at the clinic and before consultation with a clinician. The computerised record during triage does not specify the type of symptoms identified at triage. When seen by a clinician, women are asked to estimate the date of their LNMP – the first day of menstruation in their last normal menstrual cycle; this is not client-entered data. Recording date of LNMP has been routine at MSHC since 2011. Clients returning within three months of their initial visit (e.g. for results) do not have this data entered.

MSHC policy is to offer chlamydia testing to all new clients unless they have been tested recently elsewhere or are not sexually active. Endocervical, high vaginal or first-pass urinary samples are collected by healthcare workers for chlamydia testing. High vaginal samples are taken by insertion of a cotton swab marked with a prescribed depth for insertion into the vagina. In general, asymptomatic women are not examined and instead have high vaginal or urinary samples taken. Sex workers are always examined but generally have high vaginal samples as they have only speculum examinations annually if asymptomatic and our previous research has shown sex workers to be at very low risk of chlamydia [9]. Symptomatic women (and sex workers annually) are examined and have speculum examinations. This policy was consistent throughout the study period. Computerised records for all women who attended MSHC between March 2011 and 31^st^ December, 2012 were included in the data, provided they were tested for chlamydia and had a valid recorded date (0–28 days) of LNMP.

### Laboratory Methods

All specimens were analysed for *Chlamydia trachomatis* using BD ProbeTec Strand Displacement Amplification (Becton, Dickinson and Company, Sparks, MD, USA). Positive urine samples and positive swabs in BD transport medium were extracted with the MagNA Pure LC automated system (Roche Diagnostics, Indianapolis, IN). The *C.trachomatis* genome copy number was determined for each DNA extract by 1PCR [10] in conjunction with a quantified *C.trachomatis* standard (Advanced Biotechnologies Inc. (ABI), Columbia, MD). Beta globin gene qPCR was used to assess sample adequacy as well as to measure sampling variability between participants and swabs by correlation with the number of eukaryotic cells collected, according to a described protocol [11].

### Data Exclusion

Women who did not have a valid recorded date of LNMP, and/or were not tested for chlamydia during the study period were excluded from the analysis. Women tested only for anal chlamydia were also excluded. The clinic does not test for pharyngeal chlamydia.

The self-guided computer questionnaire does not allow clients to distinguish hormonal Intra-Uterine Devices (IUD) from non-hormonal copper IUD. Therefore, for the purposes of analysis of hormonal contraception, IUDs were excluded from analyses of hormonal versus non-hormonal contraception types.

### Analytic Methods

Univariate analysis was used to calculate crude odds ratios and 95% confidence intervals, and logistic regression was to investigate the association of time of the menstrual cycle with chlamydia adjusted for potential confounding factors. Weeks 1 and 2 (follicular phase) of the menstrual cycle were compared to Weeks 3 and 4 (luteal phase) because antecedent studies demonstrated an association of chlamydia detection with the latter half of the cycle. Analysis was stratified by current hormonal contraception use depending on whether or not clients were users of hormonal contraception (Combined Oral Contraceptive Pill (OCP), vaginal ring, Progestogen implant/injection). All analysis accounted for repeat attendance by individuals by including the patients' unique identification code as a cluster variable in the regression model.

In order to adjust for sampling variability, chlamydial load was divided by the number of eukaryotic cells (beta-globin) and expressed as the number of organisms present per 100 cells – this was the logarithm transformed for analysis. The mean and 95% CI organism load, beta-globin and chlamydial load per 100 cells were calculated for each specimen type (cervical swab, high vaginal swab and urine specimen). Least squares linear regression was used to compare organism load per 100 cells between different groups, accounting for repeated measures from individual women.

Data was analysed using SPSS v21 and Stata version 11 (Stata statistical Software: Release 11.0. College Station TX: Stata Corporation).

## Results

There were 25,769 consultations during the study period March 2011–31^st^ December, 2012, for 9,732 individual women. Of these consultations, 13,387 recorded a date of LNMP, of which 11,220 were within the valid range (0–28 Days). Of these 11,220 consultations, 10,017 had a chlamydia test undertaken on the day of consultation and were included in the analysis (5,055 individual women). There were 417 consultations with a diagnosis of chlamydia. Chlamydia was detected in 69 of 4,198 (1.6%) high vaginal samples, 228 of 4,368 (5.3%) cervical samples and 126 of 1,520 (10.1%) first-pass urinary (FPU) samples.

Overall, 33% (n = 3,345) were first-time consultations and 67% (n = 6,672) had previously attended MSHC. The mean age of women included was 29.9±7.4 years and 32% (n = 3,164) of consultations were for women ≤25 years of age and 68% (n = 6,853) of consultations were for women >25 years of age.

Among the 10,017 consultations, the mean day of presentation in the menstrual cycle (± standard deviation) was day 13 (13.0±7.9 days).

### Relation of Chlamydia Infection to Days since LNMP

Table 1 shows the proportion of chlamydia tests positive at different weeks within the cycle. Univariate analysis found that chlamydia infection was significantly associated with the luteal phase (Days 15–28) of the menstrual cycle (crude OR 1.3, 1.0–1.6, p = 0.012). After adjusting for other factors (age ≤25 years, presence of symptoms, number of male sexual partners, condom use, contact with an overseas sexual partner, hormonal contraception and specimen types), multivariate analysis found that chlamydia was significantly more likely to be diagnosed the luteal phase of the menstrual cycle, compared with the follicular stage (adjusted (a) OR 1.4, 95% CI: 1.1–1.8). (Table 2)

**Table 1:** Number of chlamydia tests and diagnoses by weeks of the menstrual cycle.

	All Women n/N (%)	Women using hormonal contraception n/N (%)	Women not using hormonal contraception n/N (%)
Week 1 (Days 0–7)	128/3220 (4.0%)	51/719 (7.1%)	41/957 (4.3%)
Week 2 (Days 8–14)	105/2966 (3.5%)	47/632 (7.4%)	32/1056 (3.0%)
Week 3 (Days 15–21)	114/2332 (4.9%)	46/514 (8.9%)	52/857 (6.1%)
Week 4 (Days 16–28)	70/1499 (4.7%)	26/325 (8.0%)	27/525 (5.1%)
**P-value** *	0.065	0.664	0.012
Weeks 1 and 2	233/6186 (3.8%)	98/1351 (7.3%)	73/2013 (3.6%)
Weeks 3 and 4	184/3831 (4.8%)	72/839 (8.6%)	79/1382 (5.7%)
**P-value** **	0.012	0.259	0.004
**Crude OR (95% CI)** (Reference: Weeks 1 and 2)	1.3 (1.1–1.6)	1.3 (0.9–1.7)	1.6 (1.2–2.2)

N = number tested, n = number positive, % refers to percentage positive OR = odds ratio.

* For difference in proportion between the four weeks.

** For difference in proportion between weeks 1 and 2 compared to weeks 3 and 4.

**Table 2:** Risk Factors for Chlamydia detection (All women).

	Total Consults	Number Positive (%)	Crude OR (95% CI)	P-value	Adjusted OR (95% CI)	P-value
**Menstrual Week**						
≤2 Weeks (0–14 Days)	6186	233 (3.8%)	1		1	
>2 Weeks (15–28 Days)	3831	184 (4.8%)	1.3 (1.1–1.6)	0.013	1.4 (1.1–1.8)	0.004
**Age**						
≤25 years	3164	234 (7.4%)	2.9 (2.4–3.9)	<0.001	1.6 (1.2–2.1)	<0.001
>25 years	6853	183 (2.7%)	1		1	
**Symptoms**						
Presence	2970	164 (5.5%)	1.5 (1.2–1.9)	<0.001	0.9 (0.7–1.1)	0.220
Absence	6140	232 (3.8%)	1		1	
**# Male Sexual Partners in the preceding 12 months**						
0–2	6095	152 (3.0%)	1		1	1
≥3	2586	225 (8.7%)	3.1 (2.5–3.9)	<0.001	1.7 (1.2–2.2)	<0.001
**Condom Use with Male Sexual Partners in the preceding 12 months**						
Always	1529	60 (4.0%)	1		1	
Usually/Sometimes/Never/Unsure	4453	296 (6.7%)	1.7 (1.3–2.3)	<0.001	1.5 (1.0–2.0)	0.027
**Overseas Sexual Contact/Partner from Overseas (in the last 12 months) – New Zealand excluded**						
Yes	2196	176 (8.0%)	2.3 (1.9–2.9)	<0.001	1.4 (1.1–1.8)	0.016
No	5302	192 (3.6%)	1		1	
**Hormonal Contraception Used (OCP, NuvaRing, Progestogen Implant/Injection)**						
Yes	2190	170 (7.8%)	1.8 (1.4–2.3)	<0.001	1.6 (1.3–2.0)	<0.001
No	3395	152 (4.5%)	1		1	
**Site of Sample**						
High Vaginal Swab	4165	59 (1.4%)	1		1	
Cervical Swab	4351	228 (5.2%)	2.9 (2.4–3.7)	<0.001	2.0 (1.3–3.0)	0.001
Urinary Sample	1501	120 (8.0%)	4.6 (3.7–5.8)	<0.001	2.5 (1.6–3.9)	<0.001

OR = odds ratio, CI = confidence interval.

A further analysis investigated the role of natural hormonal variations in the association between stage of the menstrual cycle and chlamydia detection. The luteal phase of the menstrual cycle was significantly associated with chlamydia detection in women not using hormonal contraception (aOR 1.6, 95% CI: 1.2–2.2, p = 0.004) but not in women taking hormonal contraception (aOR 1.3, 95% CI: 0.9–1.7, p = 0.26). (Table 3)

**Table 3:** Comparison of risk factors for chlamydia detection between hormonal contraception users and non-users.

	Women using hormonal contraception					Women not using hormonal contraception				
	Total Consults	Crude OR	P-value	Adjusted OR	P-value	Total Consults	Crude OR	P-value	Adjusted OR	P-value
**Menstrual Week**										
≤2 Weeks (0–14 Days)	1351	1		1		2013	1		1	
>2 Weeks (15–28 Days)	839	1.2 (0.9–1.7)	0.27	1.3 (0.9–1.8)	0.175	1382	1.6 (1.1–0 2.2)	0.004	1.6 (1.1–2.3)	0.007
**Age**										
≤25 years	1014	2.1 (1.5–2.2)	<0.001	1.4 (1.0–2.1)	0.027	1189	228 (1.6–3.1)1	<0.001	1.8 (1.3–2.6)	0.001
>25 years	1176	1		1		2203	1		1	
**Symptoms**										
Present	867	0.9 (0.6–1.2)	0.49	0.7 (0.6–1.0)	0.076	1353	1.3 (1.0–1.8)	0.13	1.004 (0.7–1.5)	0.98
Absent	1230	1		1		1912	1		1	
**# Male Sexual Partners in the preceding 12 months**										
0–2	1179	1		1		2031	1		1	
≥3	993	2.6 (1.9–3.6)	<0.001	1.6 (1.1–2.4)	0.010	1329	2.4 (1.8–3.4)	<0.001	1.7 (1.1–2.5)	0.012
**Condom Use with Male Sexual Partners in the preceding 12 months**										
Always	387	1		1		785	1		1	
Usually/Sometimes/Never/Unsure	1473	1.9 (1.2–3.0)	0.009	1.6 (1.0–2.5)	0.072	1963	1.7 (1.1–2.7)	0.013	1.4 (0.9–2.1)	0.18
**Overseas Sexual Contact/Partner from Overseas (in the last 12 months) – New Zealand excluded**										
Yes	769	2.1 (1.5–2.9)	<0.001	1.5 (1.1–2.2)	0.014	1048	1.7 (1.2–2.4)	0.003	1.2 (0.8–1.7)	0.38
No	1377	1		1		2221	1		1	
**Site of Test**										
High Vaginal Swab	557	1		1		896	1		1	
Cervical Swab	1093	2.7 (1.5–4.4)	<0.001	1.9 (1.0–3.2)	0.037	1798	3.1 (1.8–5.4)	<0.001	2.2 (1.2–4.2)	0.011
Urinary Sample	540	4.1 (2.4–7.0)	<0.001	2.3 (1.2–3.9)	0.008	701	4.1 (2.3–7.5)	<0.001	2.9 (1.5–5.7)	0.002

OR = odds ratio, CI = confidence interval.

### Chlamydia Bacterial Load Quantification

A total of 319 samples were available for organism load assay to quantify the bacterial load and beta-globin concentration, 43 high vaginal, 87 urinary and 189 cervical samples. Chlamydia organism load was greatest in cervical samples and smallest in urine samples (p<0.01), but chlamydia load per 100 cells was greatest for urine samples and smallest for high vaginal samples (p<0.01) When stratified by specimen type, organism load per 100 cells was not significantly different between samples taken in either the luteal or follicular stages of the cycle. (Table 4)

**Table 4:** Chlamydia and beta-globin load (All).

	Menstrual Week	Chlamydia load (log_10_)	Beta-globin load (log_10_)	Chlamydia load per 100 cells (log_10_)	P-value*
Urinary Samples (n = 87)	Weeks 1 and 2	1.04 (0.78, 1.30)	2.38 (2.12, 2.65)	0.67 (0.35, 0.99)	0.122
	Weeks 3 and 4	1.63 (1.24, 2.03)	2.59 (2.23, 2.95)	1.05 (0.68, 1.42)	
Cervical Swabs (n = 189)	Weeks 1 and 2	2.65 (2.38, 2.92)	5.06 (4.94, 5.17)	−0.44 (−0.69, −0.19)	0.951
	Weeks 3 and 4	2.80 (2.50, 3.11)	5.20 (5.08, 5.33)	−0.42 (−0.73, −0.12)	
High vaginal swabs (n = 43)	Weeks 1 and 2	1.84 (1.21, 2.48)	5.00 (4.82, 5.19(	−1.22 (−1.74, −0.70)	0.265
	Weeks 3 and 4	1.83 (1.14, 2.53)	5.43 (5,17, 5.69)	−1.70 (−2.43, −0.97)	

Chlamydia and beta-globin load (Hormonal contraception only)					
	Menstrual Week	Chlamydia load (log_10_)	Beta-globin load (log_10_)	Chlamydia load per 100 cells (log_10_)	P-value
Urinary Samples (n = 48)	Weeks 1 and 2	0.93(0.57, 1.29)	2.44 (1.86, 2.63)	0.71 (0.20, 1.22)	0.23
	Weeks 3 and 4	1.67 (1.17, 2.18)	2.42 (2.10, 2.73)	1.14 (0.62, 1.66)	
Cervical Swabs (n = 73)	Weeks 1 and 2	3.02 (2.70, 3.34)	5.09 (4.88, 5.30)	−0.16 (−0.47, 0.16)	0.97
	Weeks 3 and 4	3.10 (2.68, 3.52)	5.27 (5.04, 5.50)	−0.17 (−0.60, 0.26)	
High vaginal swabs (n = 15)	Weeks 1 and 2	1.99 (0.96, 3.03)	5.10 (4.76, 5.44(	−1.19 (−2.02, −0.35)	0.08
	Weeks 3 and 4	1.90 (0.69, 3.11)	5.95 (5.45, 6.49)	−2.04 (−3.10, −0.98)	

Chlamydia and beta-globin load (No hormonal contraception)					
	Menstrual Week	Chlamydia load (log_10_)	Beta-globin load (log_10_)	Chlamydia load per 100 cells (log_10_)	P-value
Urinary Samples (n = 35)	Weeks 1 and 2	1.20 (0.72, 1.68)	2.61 (2.15, 3.08)	0.51 (−0.01, 1.03)	0.27
	Weeks 3 and 4	1.67 (0.99, 2.34)	2.87 (2.01, 3.64)	0.92 (0.35, 1.49)	
Cervical Swabs (n = 77)	Weeks 1 and 2	2.50 (1.97, 3.03)	4.95 (4.76, 5.13)	−0.42 (−0.90, 0.06)	0.54
	Weeks 3 and 4	2.57 (2.10, 3.04)	5.21 (5.06, 5.36)	−0.64 (−1.16, −0.12)	
High vaginal swabs (n = 9)	Weeks 1 and 2	1.66 (−19.2, 23.0)	4.86 (3.85, 5.86)	−1.16 (−15.5, 13.1)	0.95
	Weeks 3 and 4	2.04 (1.00, 3.07)	5.21 (4.74, 5.67)	−1.23 (−2.63, 0.17)	

* Least squares regression comparing chlamydial load per 100 cells between weeks 1 and 2 with weeks 3 and 4 accounting for repeated measures from women.

## Discussion

In our study, we found that chlamydia detection was approximately 40% more common in the luteal phase of the menstrual cycle, and this significant association was only evident in women not using hormonal contraceptive agents. Our findings are consistent with smaller antecedent studies that demonstrated increased chlamydia detection in the latter half of the menstrual cycle in women from the general population. However in this first analysis of chlamydia loads during the menstrual cycle we were unable to find any significant association between organism load and stage of the menstrual cycle, raising questions about the mechanism by which the increased detection may occur.

Our study has a number of limitations that need to be considered. Firstly, it is a retrospective cross-sectional observational study and therefore may be subject to systematic bias. We adjusted for the predictors of chlamydia found in other studies, such as age, number of sexual partners and condom use, although it is possible that residual or unmeasured confounding remains. Secondly, not all women had the same sample collected and consistent with others, we found that the chlamydia load did vary considerably between specimen type [12]. Thirdly, not all women in our study had a speculum exam, so we were unable to comment on presence of absence of a cervical ectropion, which has been previously reported to be a risk factor for chlamydia detection [13]. Further, the presence of ectropion has been previously found to negate the association between chlamydia detection and the stage in the menstrual cycle [6]. Our questionnaire did not enquire after women's prior menstrual cycling and so our study may have included women with cycle irregularities. It must also be noted that despite date of LNMP being a clinician-administered question, women may have had difficulty in recalling their date of LNMP. Recalling date of LNMP may have therefore been a combination of women's recollection and clinical judgement, however clinicians did retain the capacity to leave dates blank if women were entirely unable to recall dates. Nevertheless, we seek to build upon studies undertaken in the 1990's that demonstrated increased chlamydia detection in the latter half of the menstrual cycle [6], [7], [14]. Our study represents the largest cross-sectional analysis to date that has addressed this potentially important variable, and provides the framework for future studies in this area. Further, a strength of our analysis was that we were able to demonstrate that the association between menstrual cycle and chlamydia detection was only significant for those not taking any hormonal contraception giving further weight to the finding.

It is possible that contamination of samples with blood may have influenced our results. The product specifications for the assay that we used, indicate that blood at a concentration of 5% volume per volume, may induce false negative results [15]. However concurrent menses has not been shown to affect the calculated sensitivity of any either cervical or urinary nucleic acid amplification for some assays, so if this was the reason for our findings it would only be applicable to laboratories that used assays influenced by concurrent blood [16].

It has been suggested that the hormonal and environmental influences on chlamydia detection is more significant in patients from groups with lower exposure to the infection such as those in our study [7]. It is possible that the failure of earlier human studies to demonstrate an association with the menstrual cycle and chlamydia infection may have been due their patient population that had an exceptionally high chlamydia prevalence of up to 33 and 35%, in studies of women that were sexual contacts of men with non-gonococcal urethritis. The higher prevalence of infection may have been associated with increased bacterial load due to recently acquired infection. In contrast, our study represents a more general cross-section of the population and demonstrated a prevalence of chlamydia infection of 4.2%, closer to the estimates of 3.4% for women in the general community [17].

There are a number of possible biological mechanisms that might be responsible for our findings. Firstly chlamydia detection may be influenced by hormone changes during the second half of the menstrual cycle when progesterone levels rise and oestrogen levels fall [18]. Secondly hormonal and possible inflammatory mediators or changes in cell structure and integrity, may influence the susceptibility of the female reproductive tract to infection at different stages in the menstrual cycle [19], [20]. Other changes seen in the post-ovulatory phase of the menstrual cycle that may modulate host responses and allow increased growth of chlamydia include an elevated ratio of protein to mucous glycoprotein in cervical mucus [21] and decreased levels of IgA and IgG in vaginal fluid and cervical mucus [22]. The cytolytic capacity of CD3+ T-cells has been shown to be reduced in the luteal phase of the menstrual cycle [23]. Many cytokines, including inflammation-associated Interleukin-8 (IL-8), have been shown to play a key role in immune responses to chlamydia [4], [24], and levels of IL-8 have been shown to be markedly lowered in the luteal phase of the menstrual cycle [25]. Any of these factors may be associated with an increasing bacterial load of chlamydia to above that of the sensitivity detection of the assay used. Investigation of these factors and further investigation of influencing hormonal factors would be beneficial in further explaining the association of higher chlamydia detection in the luteal phase of the menstrual cycle. It is important to note that we did not find significantly higher chlamydia load so if any of these factors did alter chlamydia detection, it may not be through a higher load.

There are a number of possible reasons for our finding that chlamydia was more commonly detected during the second half of the menstrual cycle, but that chlamydia load was not higher during this time. Firstly it is possible that the factors that influenced chlamydia load in the second half of the menstrual cycle, did so only for low levels of infection and were therefore not apparent in our analysis comparing mean loads. It is also possible that our analysis, which was stratified by specimen type, had limited power to detect differences in load. Our study is one of the largest to date but future studies involving larger numbers of specimens may be able to address this issue. Finally, it is possible that load does not vary during the menstrual cycle, and that our analysis of chlamydia positivity, was influenced by the factors discussed in the second paragraph of the discussion.

Our findings may have important implications for chlamydia screening policy. The success of population-based screening programs depends in part on the sensitivity of the assays used. If the sensitivity was increased by testing mainly in the luteal phase of the cycle, it may be that successful screening programs could be achieved with less frequent screening. This would, however, need to be balanced with the realistic logistic difficulties in organising screening during a specific time in the cycle, as screening for chlamydia is frequently opportunistic. Of broader interest from our work is that the physiological changes in the female genital tract that occur during the menstrual cycles may underlie varying immune reactions to chlamydia. Our findings of a higher detection rate of chlamydia in a period of the menstrual cycle known to have decreased immunity in the female genital tract may be important in the further characterisation of immune responses to chlamydia.
